# Adherence to optimal medical therapy and control of cardiovascular risk factors in patients after ST elevation myocardial infarction in Mexico

**DOI:** 10.3389/fcvm.2024.1384684

**Published:** 2024-07-23

**Authors:** Montserrat Villalobos-Pedroza, Sarai Hernandez-Pastrana, Alexandra Arias-Mendoza, Ximena Latapi-Ruiz Esparza, Mariana Robles-Ledesma, Alejandra Guerrero-Ochoa, Nelson Antonio Milanes-Gonzalez, Fabio Solis-Jimenez, Alejandro Sierra Gonzalez-De Cossio, Alejandro Pablo Flores-Batres, Arielle Astrid Brindis-Aranda, Edgar Rivera-Pedrote, Alejandra Jara-Nevarez, Eder Gonzalez-Macedo, Rodrigo Gopar-Nieto, Héctor Gonzalez-Pacheco, Jose Luis Briseño-De la Cruz, Diego Araiza-Garaygordobil

**Affiliations:** Cardiovascular Critical Care Unit, Instituto Nacional de Cardiología “Ignacio Chávez”, México City, Mexico

**Keywords:** adherence, risk factors, STEMI, optimal medical therapy, developing countries, Latin-American

## Abstract

**Introduction:**

In developing countries, there is a notable scarcity of real-world data on adherence to optimal medical therapy (OMT) and its correlation with major cardiovascular adverse events (MACEs) after ST-elevation myocardial infarction (STEMI). Our study focuses on addressing this gap by evaluating adherence to OMT, examining its influence on the risk of MACEs after STEMI, and assessing subsequent cardiovascular risk factor control in Mexico.

**Methods:**

We conducted a prospective observational study of post-STEMI patients after hospital discharge. Adherence to treatment was assessed over a median of 683 days (interquartile range: 478–833) using the Simplified Medication Adherence Questionnaire (SMAQ). Patients were followed up for 4.5 years to monitor MACEs (cardiovascular death, cardiogenic shock, recurrent myocardial infarction, and heart failure).

**Results:**

We included 349 patients with a mean age of 58.08 years (±10.9), predominantly male (89.9%). Hypertension (42.4%), smoking (34.3%), type 2 diabetes mellitus (31.2%), obesity (22.92%), and dyslipidemia (21.4%) were highly prevalent. Adherence to OMT per SMAQ was 44.7%. The baseline clinical characteristics of adherent and non-adherent patients did not significantly differ. OMT prescription rates were as follows: acetylsalicylic acid, 91.1%; P2Y12 inhibitors, 76.5%; and high-intensity statins, 86.6%. While non-adherent patients had a numerically higher rate of MACEs (73 vs. 49 first events), there was no statistically significant difference (hazard ratio 1.30, 95% confidence interval 0.90–1.88).

**Discussion:**

In this real-world study of patients after STEMI, we observed low adherence to OMT, a low proportion of global cardiovascular risk factor control, and a numerically higher incidence of recurrent major adverse cardiovascular events in non-adherent patients. Strategies to improve adherence to OMT and risk factor control are needed.

## Introduction

Adherence to guideline-indicated therapy following ST-elevation myocardial infarction (STEMI) is a crucial yet often overlooked aspect of patient care. Studies consistently demonstrate its direct impact on reducing the risk of major adverse cardiovascular events (MACEs). Nevertheless, data report that at least 50% of patients are non-adherent to their prescribed therapy within the first 2 years after their index event (1). Optimal medical therapy (OMT) after STEMI includes a combination of antiplatelet agents, beta-blockers, angiotensin-converting enzyme inhibitors (ACEIs) or angiotensin receptor blockers (ARBs), and lipid-lowering therapies; these have been shown to significantly reduce mortality and morbidity (2). In resource-limited countries like Mexico, achieving therapeutic compliance—the extent to which patients adhere to their prescribed medications and treatment regimens—can be challenging. Various factors, including socio-economic, cultural, healthcare system-related, and patient-related influences, collectively impact the likelihood of adherence to the recommended medical regimens (3, 4). Amidst these challenges, a further essential objective emerges—optimizing cardiovascular risk factor control after STEMI to improve long-term prognosis.

This study aimed to (1) assess adherence to OMT using the Simplified Medication Adherence Questionnaire (SMAQ) in patients after STEMI, (2) evaluate the proportion of patients who achieved optimal control of cardiovascular risk factors during follow-up, and (3) determine the time to first occurrence of major adverse cardiovascular events (MACEs) based on the presence (or lack) of adherence to OMT.

## Materials and methods

### Study design and population

The population of the present study is derived from PHASE-Mx (PHArmacoinvasive Strategy vs. primary PCI in STEMI: a prospective registry in a largE geographical area) (www.clinicaltrials.gov, NCT03974581); the description, design, scope, and detailed results of the PHASE-MX study have been published elsewhere (5, 6). This prospective observational study included adults over 18 years old diagnosed with STEMI who received reperfusion treatment within the first 12 h of symptom onset.

For the present analysis, we included patients who were discharged alive after index hospitalization. STEMI was defined as ST-segment elevation higher than 1 mm in two consecutive anatomical electrocardiogram leads (7). The present study complies with ethical standards and the principles outlined in the Declaration of Helsinki.

### Data acquisition

Demographic data and baseline clinical indicators were collected from the electronic medical records of the study center, including pertinent medical history, initial laboratory workup, in-hospital treatment, and reperfusion strategy. Participants who met the inclusion criteria were reached via phone for initial inquiries to confirm their survival status. Consent to participate in the study was sought from the participants or their caregivers. For deceased patients, respondents were asked to provide the date and cause of death. If consent was approved, adherence to treatment was assessed using the SMAQ over a median of 683 days [interquartile range (IQR): 478–833] after STEMI. The SMAQ is a validated psychometric tool initially developed to assess adherence to antiretroviral therapy in human immunodeficiency virus-positive patients; it has since been validated in Spanish to evaluate adherence to medication in chronic diseases. The SMAQ includes the following questions: (1) Do you forget to take your medicine? (2) Are you careless at times about taking your medicine? (3) Sometimes, if you feel worse, do you stop taking your medicine? (4) Thinking about last week, how often have you not taken your medicine? (5) Did you not take any of your medicine over the past weekend? and (6) Over the past 3 months, how many days have you not taken any medicine at all? Non-adherence was determined if the patient answered “Yes” to questions 1, 3, or 4; or “No” to question 2; reported “more than two missed doses” in question 5; or reported “more than two days in the previous three months” in question 6. Adherence was determined if the opposite responses were given (8, 9).

Medication prescription and usage were also assessed, including acetylsalicylic acid, P2Y12 inhibitors, beta-blockers, angiotensin-converting enzyme inhibitors (ACEIs) or angiotensin receptor blockers (ARBs), lipid-lowering therapies (type and dosage of statins, ezetimibe, and proprotein convertase subtilisin/kexin type-9 inhibitors), and antidiabetic medications for patients diagnosed with diabetes (such as metformin, sodium-glucose transport protein 2 inhibitors, glucagon-like peptide-1 agonists, sulfonylureas, dipeptidyl peptidase 4 inhibitors, and other therapies).

Cardiovascular risk factor control was evaluated using the care goals provided by the 2021 European Society of Cardiology Guidelines on Cardiovascular Disease Prevention and the updated 2023 European Society of Cardiology Acute Coronary Syndrome Guidelines. We assessed systolic blood pressure, diastolic blood pressure, smoking status, total cholesterol, high-density lipoprotein cholesterol (HDL-C), non-HDL-C, low-density lipoprotein cholesterol (LDL-C), triglycerides, and hemoglobin A1C (HbA1C) (10, 11).

Finally, the occurrence of major cardiovascular events during follow-up was evaluated using electronic medical records and, in cases of uncertainty, via phone calls. Every effort was made to collect information on all clinical events; patients with uncertain or inaccessible information were excluded from the analysis.

Adherence to OMT was defined according to the SMAQ questionnaire, as previously reported, and according to patients’ responses to questions 1–6. Optimal control of cardiovascular risk factors during follow-up was assessed during the first scheduled visit after STEMI hospital discharge and evaluated dichotomously following the updated 2021 European Society of Cardiology Guidelines on Cardiovascular Disease Prevention and the 2023 European Society of Cardiology Acute Coronary Syndrome Guidelines (LDL-C controlled if <55 mg/dl; HbA1C controlled if <7.0%, blood pressure controlled if <130/80 mmHg at the time of assessment; smoking controlled if the patient reported no smoking since hospital discharge) (10, 11).

Major adverse cardiovascular events were evaluated using a composite endpoint comprising death from cardiovascular causes, congestive heart failure, recurrent myocardial infarction (MI), and cardiogenic shock. The components of the primary composite outcome were defined according to the 2017 Cardiovascular and Stroke Endpoint Definitions for Clinical Trials (12). Patients who could not be contacted due to lack of contact information, non-response to phone calls, refusal to participate in the survey, or inability to reach their family members were excluded from the study.

### Statistical analysis

For analysis of continuous variables, the Shapiro–Wilk test was used to determine whether variables were normally distributed; normally distributed variables are presented as mean ± standard deviation, while non-normally distributed variables are presented as medians and interquartile ranges. The Mann–Whitney *U* test was used for statistical comparisons of continuous variables when the null hypothesis of normality was rejected. Categorical variables were described with frequencies and percentages. The chi-squared test and Fisher's exact test were used for statistical comparisons of categorical variables. For analysis of the primary endpoint of adverse cardiovascular events, the differences in time to the event of interest between groups were calculated using the log-rank test and depicted using cumulative event Kaplan–Meier curves; additionally, the Cox regression model assessed the actual effect of non-adherence on MACEs. For all analyses, a *p*-value <0.05 was considered statistically significant. Statistical analysis was performed using STATA v14.1 (StataCorp LP, College Station, TX, USA).

## Results

### Patient population

**Figure 1:**
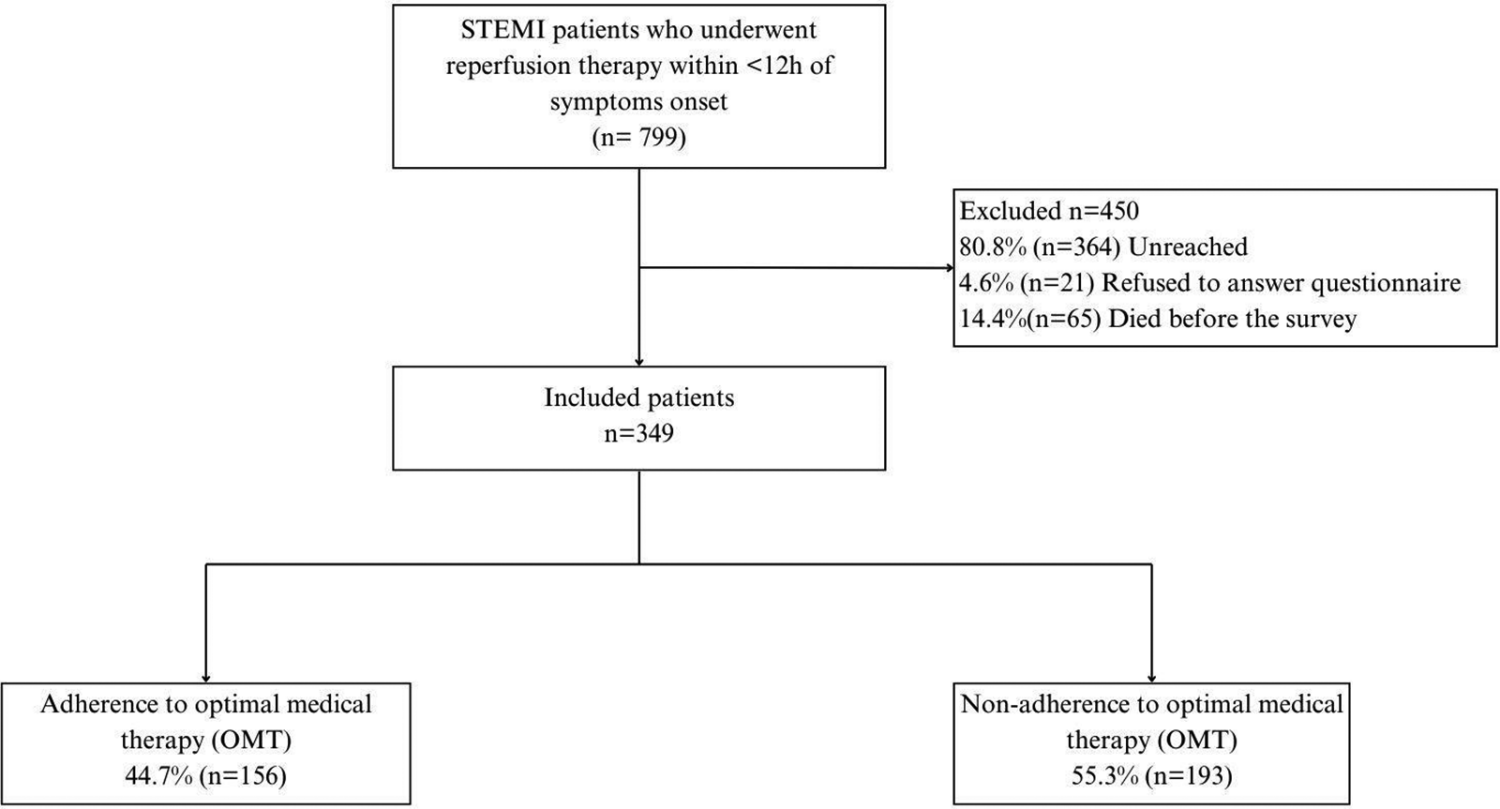
Study flowchart.

From April 2018 to November 2021, 799 patients with STEMI who received reperfusion therapy within 12 h since symptom onset and were discharged alive were identified as the initial sample. Following initial and subsequent attempts to contact the patient via phone call (as per protocol), 450 patients were excluded (Figure 1). The main reason for excluding patients was the inability to contact them after multiple attempts (80.8%). Ultimately, the final analytical sample consisted of 349 patients.

The mean age of presentation was 58.08 years (±10.9), and the majority were male (89.9%). The most prevalent cardiovascular risk factors were hypertension (42.4%), smoking (34.3%), type 2 diabetes mellitus (31.2%), obesity (22.92%), and dyslipidemia (21.4%). Regarding reperfusion strategy, 183 patients (52.4%) underwent primary percutaneous coronary intervention (pPCI), while 166 patients (47.6%) underwent a pharmacoinvasive strategy (thrombolysis + pPCI). Table 1 includes a comprehensive list of baseline clinical features.

**Table 1:** Baseline characteristics according to adherence to OMT.

	Adherence (*n* = 156)	No adherence (*n* = 193)	*p*-value
Demographic characteristics			
Age, mean	58.7 ± 11.01	57.5 ± 10.8	0.31
Male, *n* (%)	137 (87.82)	177 (91.71)	0.22
Clinical presentation			
Hemoglobin, g/dl	15.69 ± 1.63	15.51 ± 1.96	0.35
Leukocytes, 10^3^/µl	11.84 ± 3.36	11.56 ± 3.45	0.44
Glucose, mg/dl	188.66 ± 91.5	183.22 ± 93.28	0.58
Blood urea nitrogen, mg/dl	19.17 ± 9.58	19.26 ± 9.58	0.93
Hs-cTnI, pg/ml	9.5 (0.8–35)	16.1 (0.5–71)	0.17
Hs-cTnI peak, pg/ml	57 (20.3–80)	68.15 (24.3–80)	0.70
NT-proBNP, pg/ml	206 ± 41	213 ± 372	0.46
PCR, mg/dl	25.73 ± 56.59	30.88 ± 56.70	0.40
Na, mmol/L	135 ± 3.3	135 ± 2.9	0.63
Cl, mmol/L	102.92 ± 4.0	103.40 ± 3.42	0.23
Alb, g/dl	3.67 ± 0.43	3.69 ± 0.48	0.62
Cholesterol, mg/dl	163.16 ± 44.19	161.62 ± 41.72	0.74
LDL-C, mg/dl	101.04 ± 37.53	101.44 ± 38.14	0.92
HDL-C, mg.dl	35.54 ± 9.35	36.45 ± 12.15	0.45
HbA1C, %	6.86 ± 1.98	7.17 ± 2.17	0.10
Comorbidities, *n* (%)			
Hypertension	71 (45.51)	77 (39.90)	0.29
Diabetes mellitus	48 (30.77)	61 (31.61)	0.86
Hypercholesterolemia	30 (19.23)	45 (123.32)	0.35
Current smoker	24 (34.78)	22 (33.85)	0.90
Obesity	36 (23.08)	44 (22)	0.95
Chronic kidney disease	5 (3.21)	3 (1.55)	0.30
Medical history, *n* (%)			
Myocardial infarction	18 (11.54)	15 (7.77)	0.23
Previous PCI	11 (7.05)	12 (6.22)	0.75
Coronary bypass graft	5 (3.21)	2 (1.04)	0.15
Known heart failure	1 (0.64)	2 (1.04)	0.69
Valvulopathy	0 (0.0)	1 (0.52)	0.36
Atrial fibrillation	1 (0.0)	2 (1.04)	0.20

### Adherence and persistence to OMT

After assessment using the SMAQ tool, 156 patients (44.7%) were categorized as adherent to OMT and 193 patients (55.3%) were categorized as non-adherent. The reasons for non-adherence are as follows: Does the patient forget to take their medications? (yes: 33.8%); Does the patient take their medications at the indicated time? (no: 24.9%); Has the patient ever stopped their medications if feeling ill? (yes: 12.0%); Did the patient forget to take their medications during the weekend? (yes: 20.3%); During the last week, how many times did the patient skip a dose? (≥three times: 6.3%); During the last 3 months, how many days has the patient completely stopped medications? (>2days: 23.2%). We assessed whether factors such as age, state of residence, socio-economic status, job, and education level might influence the lack of adherence; however, we found no statistically significant predictors. Supplementary Table 1 includes the results of a linear regression model for predicting the lack of adherence.

During the survey, patients were asked about the reason for not taking their medications properly. Among the 349 patients surveyed, 93 (26.6%) reported not taking their medications properly (compared with the actual 55.3% categorized as non-adherent using the SMAQ tool). The cited underlying reasons included the following: forgetting to take medications (80, 86.0%); not understanding the prescription (1, 1.08%); unwillingness to take medications (7, 7.53%); and side effects (5, 5.38%). No patients stated that a lack of purchasing power was a reason for poor adherence.

### Cardiovascular risk factor control during follow-up

**Figure 2:**
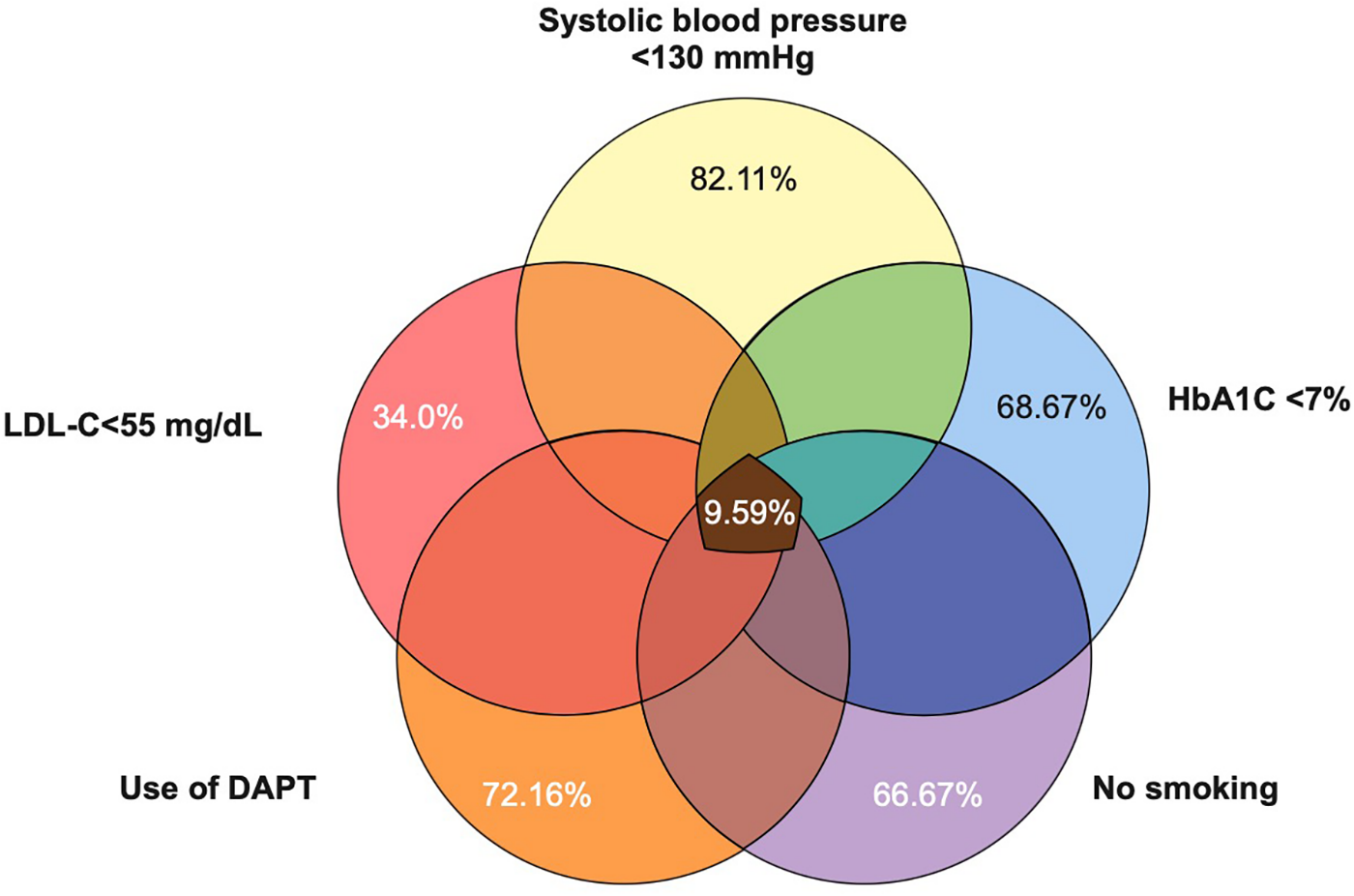
Venn diagram of control of cardiovascular risk factors.

Figure 2 (central panel) shows the proportion of patients with optimal control of cardiovascular risk factors according to the ESC 2021 Cardiovascular Prevention Guidelines and the ESC 2023 Acute Coronary Syndrome Guidelines. Of note, the proportion of patients who achieved “total control” (simultaneous control of LDL-C, use of dual antiplatelet therapy, systolic blood pressure control, HbA1C control, and absence of smoking) was 9.59%; LDL-C <55 mg/dl was the least frequently achieved individual objective (10, 11).

**Figure 3:**
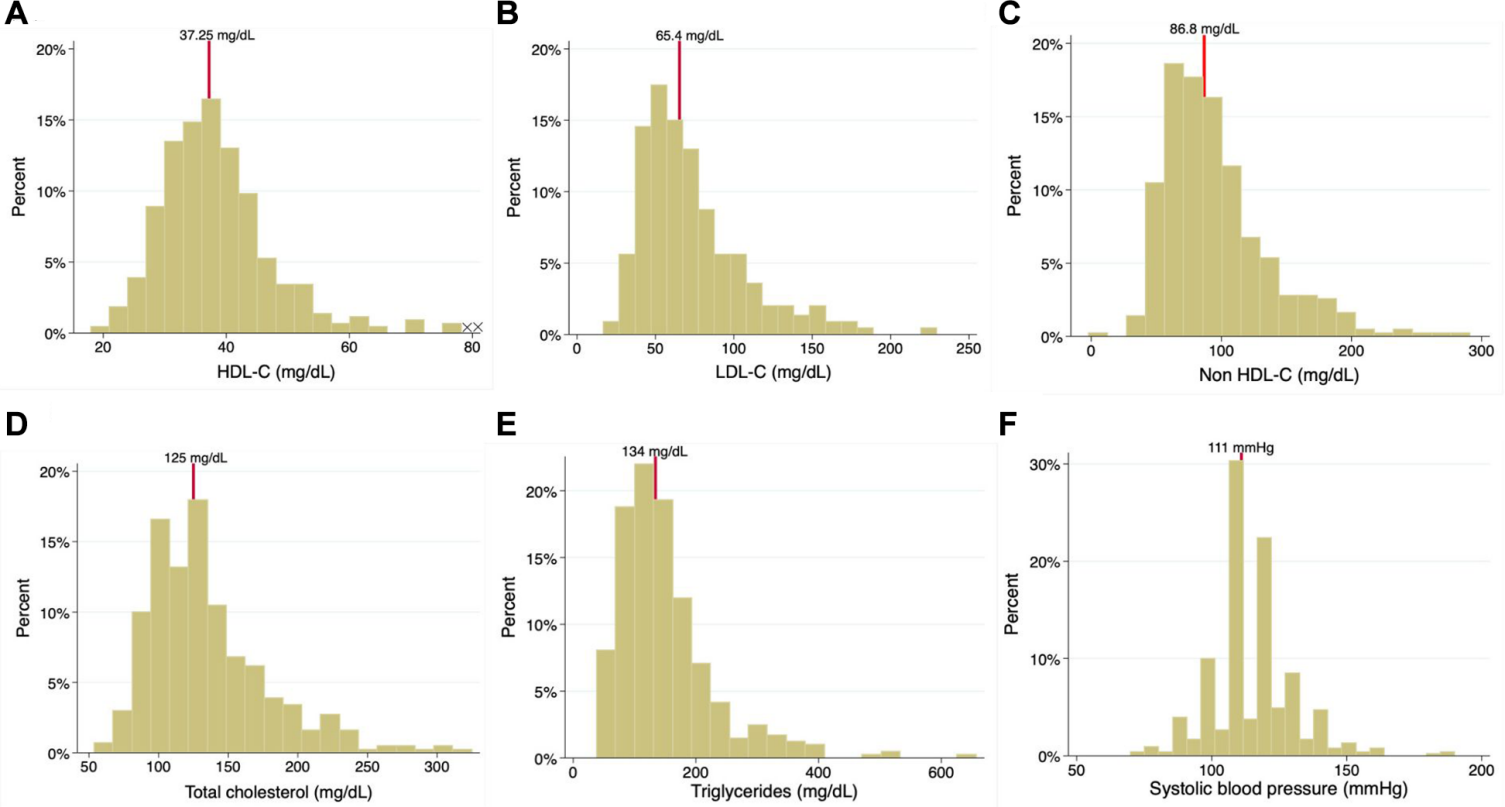
Histogram of (**A**) HDL-C, (**B**) LDL-C, (**C**) non HDL-C, (**D**) total cholesterol, (**E**) triglycerides, and (**F**) systolic blood pressure.

Median values were as follows: LDL-C, 65.4 (49.2–87.7) mg/dl; HDL-C, 37.25 (32.65–42.75) mg/dl; non-HDL-C, 86.8 (66.8–113) mg/dl; total cholesterol, 125 (104.9–144.15) mg/dl; triglycerides, 134 (98.2–179.5) mg/dl; and systolic blood pressure, 111 (110–120) mmHg. The distribution of continuous variables (total cholesterol, LDL-C, triglycerides, HDL-C, non-HDL-C, and systolic blood pressure) during follow-up is depicted in Figure 3. Table 2 presents the prescription of evidence-based therapies in the context of secondary prevention according to the adherence status (presence or lack of adherence). Of note, adherent patients were more often prescribed aspirin, P2Y12 inhibitors, statins, and ACEI/ARBs.

**Table 2:** Prescribed medication rates in patients.

	Total population (*n* = 349)	Adherence (*n* = 156)	Non-adherence (*n* = 193)	*p*-value
Prescribed medications (%)				
Aspirin	91.1	95.5	87.6	0.009
P2Y12 inhibitors	76.5	84.0	70.5	0.003
High-intensity statins	92.59	94.06	91.30	0.440
ACEI/ARB	81.4	87.9	76.2	0.005
Beta-blockers	68.5	72.5	65.3	0.153
Spironolactone	21.0	17.3	23.8	0.136
Diuretics	19.2	18.6	19.7	0.795
	Total population with diabetes (*n* = 109)	Adherence (*n* = 48)	Non-adherence (*n* = 61)	*p*-value
Antidiabetic agents (%)				
Metformin	63.9	54.3	73.0	0.099
Sulphonylureas	8.4	8.6	8.3	0.971
*Dipeptidyl peptidase*-*4* inhibitors	5.6	5.7	5.6	0.977
Sodium-glucose cotransporter-2 inhibitors	2.8	2.9	2.8	0.984
Glucagon-like peptide 1 *agonists*	0	0	0	0

### Adherence to OMT and risk of subsequent cardiovascular events

**Figure 4:**
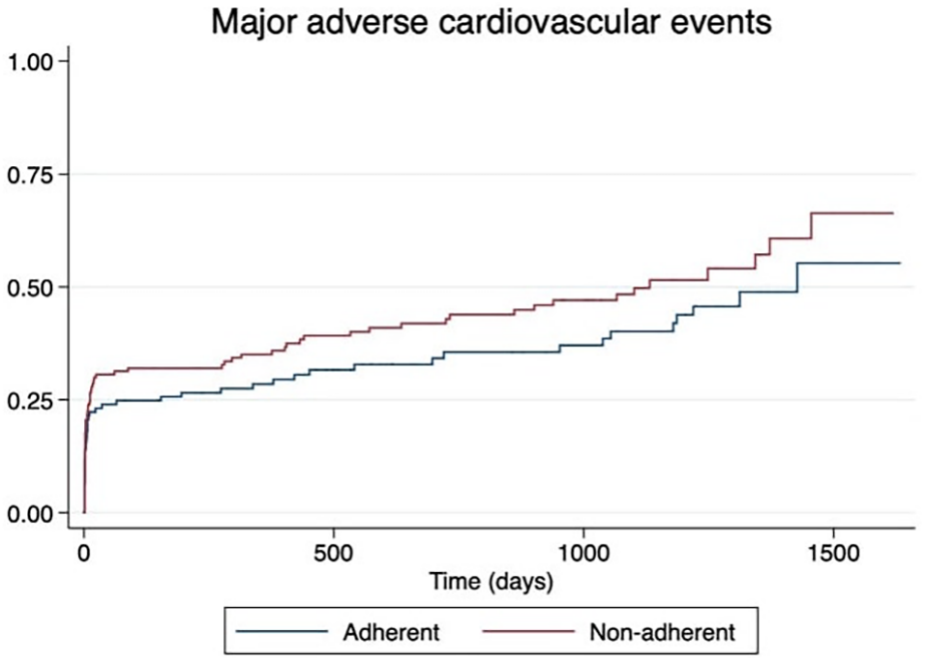
Kaplan–Meier curve estimates of the composite endpoint of cardiovascular death, cardiogenic shock, recurrent myocardial infarction, and heart failure at long-term follow-up.

The mean follow-up time after the index event was 1,613 days (4.4 years). We observed an increased number of recurrent major adverse cardiovascular events in non-adherent patients [47 patients (30%) in the non-adherent group vs. 37 patients (20%) in the adherent group]; however, this trend did not reach statistical significance [hazard ratio (HR): 1.30; 95% confidence interval (CI): 0.90–1.88] (Figure 4). The composite endpoint risk was 25.2% during the first 365 days after STEMI. Male sex (HR: 1.59, 95% CI: 1.07–2.36) was the only statistically significant variable that predicted worse outcomes in our patient population.

## Discussion

In this real-world registry conducted in Mexico, we found that less than half of our cohort was adherent to their prescribed medical therapy. Previously, the TEXTMEDS study included patients who presented with acute coronary syndrome from 13 urban and 5 rural settings in Australia; patients allocated to the control group self-reported their adherence to medications for secondary prevention (ACEI/ARB, aspirin, beta-blockers, statins, and second antiplatelets) at 6 and 12 months, showing a 54.3% adherence to all five medication classes. This contrasts with the lower adherence rate (44.7%) found in our study. However, it is relevant to note that the instrument used to evaluate adherence differed between studies; adherence in the TEXTMEDS study was measured by asking participants to self-report the number of days in the past 30 days they missed medication (13). On the other hand, the PURE study assessed the use of secondary prevention drugs for coronary heart disease in high-income, upper-middle-income, low-middle-income, and low-income countries, indicating that adherence to secondary prevention medication is suboptimal globally, with drug use rates decreasing with declining country economic wealth (14). Despite Mexico being classified as an upper-middle-income country by the World Bank, our study indicated low adherence to OMT, although no statistical significance with socio-economic status was found.

In a recent study conducted in Latin America evaluating achievement of secondary prevention goals at 12 months after an acute coronary syndrome index event, stringent goals were met by only 5.5% of the 468 patients. These goals encompassed weight loss ≥10% in patients with a previous body mass index ≥25 kg/m^2^ or maintenance of a previous body mass index ≤24.9 kg/m^2^, systolic/diastolic blood pressure <130/80 mmHg, LDL-C <55 mg/dl, and HbA1c <7%. Notably, the author's definition of stringent goals did not include smoking cessation and the use of dual antiplatelet therapy. Similarly, low rates of cardiovascular risk factor control were observed in our study, with LDL-C <55 mg/dl being the least achieved goal, met by only 22.4% of the population (15).

Evidence shows that lower LDL-C levels after presenting acute coronary syndrome are associated with reduced cardiovascular event rates, necessitating initiation of high-intensity statins at the highest tolerated dose to reach the LDL-C goals. Results from the ESC-EORP EUROASPIRE V survey, conducted across 130 centers in 27 countries among patients with coronary heart disease after hospitalization, demonstrated LDL-C ≥1.8 mmol/L in 71% and LDL-C ≥2.6 mmol/L in 33%, during a median time between hospital discharge and interview of 1.12 years (16). Regarding LDL-C lowering drug therapy, 49.9% were prescribed high-intensity statins. In our population, LDL-C lowering therapy with high-intensity statins was prescribed to 92.59%; however, only 19% of our total population achieved an LCD-C <55 mg/dl.

A study that included patients after myocardial infarction and evaluated the association between medication adherence and adverse cardiovascular events found that adherence to dual antiplatelet therapy, lipid-lowering drugs, and ACE inhibitors/ARB was significantly associated with a reduced likelihood of mortality. Furthermore, the probability of MACEs was 45% lower in patients who were adherent to lipid-lowering therapy than in non-adherent patients (17).

Our study included a broader range of medications when determining adherence, resulting in numerically higher MACEs, although the increased risk in the non-adherent group was not statistically significant. However, we observed a higher 1-year MACE rate in our population compared to other long-term series, such as the SWEDE-HEART registry (18). We hypothesize that this is due to the higher risk profile and worse prognosis in our population, which experiences longer ischemic times and lower reperfusion rates in Mexico, as reported in other studies (6, 19).

While findings from our study provide valuable insights, it is important to acknowledge certain limitations that could impact the validity of our results. First, the sample size limits the external validity of our findings. In addition, the evaluation of OMT adherence considered all prescribed medications, without assessing adherence to individual medications in relation to MACEs. Factors influencing short-term adherence may differ from those influencing long-term adherence; thus, our findings may not be generalizable to longer periods. Adherent participants were found to have higher prescription rates of aspirin, P2Y12, and ACEI/ARB, and this could represent a confounding factor in assessing OMT adherence. Efforts to improve therapeutic adherence through patient education, healthcare provider training, and the development of structured long-term programs are urgently needed to reduce the global burden of cardiovascular disease. The findings from our study should encourage the implementation of measures to improve risk factor control, with a particular emphasis on LDL-C control.

In conclusion, in this real-world study of patients after STEMI, we found low adherence to OMT, a low proportion of global cardiovascular risk factor control, and a numerically higher incidence of recurrent major adverse cardiovascular events in non-adherent patients. Our results highlight the need for increased efforts to improve adherence and access to evidence-based therapies after STEMI.

## Data Availability

The raw data supporting the conclusions of this article will be made available by the authors without undue reservation.
