# Supplementary material for: Adherence to optimal medical therapy and control of cardiovascular risk factors in patients after ST elevation myocardial infarction in Mexico
Source: Front Cardiovasc Med. 2024 Jul 23;11:1384684. doi: 10.3389/fcvm.2024.1384684 (PMC11304054; doi:10.3389/fcvm.2024.1384684)
Supplement: Supplementary file 1 [file Table1.docx]

**Supplementary material**

Table 3. Linear regression model for the prediction of the lack of adherence to OMT.

|  | Coefficient | p-value | 95% CI |
| --- | --- | --- | --- |
| Age | -.003 | 0.23 | -0.008 - 0.002 |
| Educational level | -.021 | 0.38 | -0.070 - 0.026 |
| Socioeconomic status | -.006 | 0.76 | -0.051 - 0.038 |
| Job | +.003 | 0.46 | -0.004 - 0.010 |
| Place of residency | -.017 | 0.08 | -0.036 - 0.002 |
